# Examining the association between vigilance and mind wandering

**DOI:** 10.3389/fcogn.2025.1577053

**Published:** 2025-09-01

**Authors:** Brooke Schwartzman, Anthony P. Zanesco, Ekaterina Denkova, Jason S. Tsukahara, Amishi P. Jha

**Affiliations:** ^1^Department of Psychology, University of Miami, Coral Gables, FL, United States; ^2^Department of Psychology, University of Kentucky, Lexington, KY, United States

**Keywords:** vigilance decrement, mind wandering, sustained attention, time-on-task, growth curve modeling

## Abstract

There has been a growing interest in the relationship between the vigilance decrement, characterized by performance decline with greater time-on-task, and the occurrence of mind wandering—task-unrelated thought. Recent evidence from a large-scale military sample suggests a link between performance declines and increased mind wandering over a 20-min Sustained Attention to Response Task. Herein, we examined if similar patterns are present when the task duration is shorter and delivered online to college students who rely on sustained attention for academic success. Specifically, we explored the relationship between the vigilance decrement and mind wandering in undergraduates (*N* = 310) completing a 10-min Sustained Attention to Response Task embedded with mind wandering probes. Bivariate growth curve modeling was used to examine within-task changes in performance and mind wandering over time-on-task as well as their covariance. The results revealed that a decrease in accuracy and an increase in response time variability were associated with an increase in mind wandering with greater time-on-task. In addition, self-reported task motivation, interest, and difficulty ratings were assessed as potential person-level moderators of changes with time-on-task. The results showed that individuals with higher motivation and interest ratings demonstrated a reduced time-on-task effect on response time variability and mind wandering. These findings suggest that mind wandering contributes to the vigilance decrement, even in shorter-duration tasks. Additionally, higher task-related motivation and interest appear to reduce the performance costs of mind wandering.

## 1 Introduction

The ability to sustain attention and maintain vigilance is essential for many daily tasks and activities. Despite its importance, lapses in attention are frequent and performance is known to decline with greater time-on-task, a phenomenon known as the vigilance decrement. Vigilance has historically been studied in high stakes applied contexts, such as in military relevant tasks like radar monitoring (Mackworth, 1948), where lapses in attention can lead to significant consequences. However, it is equally critical for individuals across a variety of circumstances, such as college students who rely on sustained attention to learn effectively and perform well in the classroom (Kane et al., 2021). Understanding the factors that influence the vigilance decrement is therefore important for promoting performance success across different populations and task contexts. Extrinsic factors related to the demand and information processing requirements of the task have been shown to moderate vigilance (Grubb et al., 1995; Gartenberg et al., 2018). Similarly, intrinsic factors, such as stress, motivation, and individuals' tendency to engage in mind wandering—the occurrence of task-unrelated thoughts—also influence performance and contribute to interindividual differences in the magnitude of the vigilance decrement (Brosowsky et al., 2023; Robison and Nguyen, 2023; Zanesco et al., 2024a).

The vigilance decrement, typically seen as a monotonic decline in accuracy over time-on-task, has been demonstrated in a wide variety of cognitive tasks requiring sustained attention that last from minutes to hours (See et al., 1995). In addition to within-task decreases in accuracy, several other behavioral and self-reported psychological phenomena have been examined as correlates of attentional lapses. For instance, tasks that demonstrate a vigilance decrement also show patterns of increasing response time variability (Anderson et al., 2021; Wang et al., 2014; Laflamme et al., 2018; Zanesco et al., 2013, 2018, 2020, 2024a) and increasing mind wandering with greater time-on-task (Cunningham et al., 2000; Brosowsky et al., 2023; Krimsky et al., 2017; McVay and Kane, 2012; Thomson et al., 2014; Welhaf and Banks, 2024; Zanesco et al., 2020, 2024a,b). Such patterns have led to recent theoretical accounts attempting to incorporate these phenomena into explanations of vigilance, with some proposing that changes in behavioral task performance and increasing rates of mind wandering result from the same neurocognitive mechanisms. Specifically, these theories suggest that more frequent shifts in attention from external tasks to internal thoughts—more frequent episodes of mind wandering—may underlie the vigilance decrement and occur due to failures in cognitive control (Thomson et al., 2015) or because mind wandering is more rewarding than exerting effort on a demanding, monotonous task (Kurzban et al., 2013).

Despite many studies demonstrating the occurrence of the vigilance decrement and increased rates of mind wandering in continuous performance tasks, few studies have provided direct evidence linking these patterns. Thomson et al. (2014) conducted one notable study demonstrating that within-task change-over-time rates in mind wandering were associated with within-task changes in accuracy. More recently, evidence from a large-scale study in military service members suggested that within-task increases in mind wandering were strongly correlated with decreases in performance over a 20-min continuous performance task (Zanesco et al., 2024a).

While prior evidence links within-task increases in rates of mind wandering with the vigilance decrement, it is important to examine whether these patterns also emerge in tasks of shorter duration. Indeed, researchers are often faced with limited time to administer cognitive tasks and may need to rely on the convenience of online task administration. Shortened variants of questionnaires are often employed when abbreviated variants are needed in time-pressured studies (Stanton et al., 2002). Similarly, shortened versions of widely utilized cognitive tasks have been developed and validated for both in-person and online settings (e.g., Burgoyne et al., 2023). While the vigilance decrement can emerge even in shortened continuous performance tasks (See et al., 1995), not all tasks consistently evince a vigilance decrement (i.e., Botella et al., 2001). Further, while some studies report consistent time-on-task effects across in-person and abbreviated online versions (e.g., Gyles et al., 2023; McCarley and Yamani, 2021), others have found discrepancies between formats (e.g., Thomson et al., 2016; Claypoole et al., 2018). To that end, we aim to replicate the approach used during in-person administration of the 20-min Sustained Attention to Response Task (SART) variant described by Zanesco et al. (2024a), adapting it to a 10-min online format to evaluate its validity under conditions of time pressure and online delivery. Specifically, this study builds on prior work by examining whether key performance and mind wandering dynamics replicate in a shortened online task, shedding light on the viability of assessing the vigilance decrement in time-limited, remote contexts.

The current study evaluated performance and mind wandering over the course of a 10-min continuous performance task administered online. Using bivariate growth curve modeling, the relationship between within-task changes in attentional performance and mind wandering, indexed via embedded experience sampling probes, was examined in undergraduates. This approach uniquely assesses the covariance between changes in behavioral performance and mind wandering over time-on-task. Additionally, individual differences in self-reported task motivation, interest, and difficulty were examined to determine if they relate to changes in performance and mind wandering over time. In line with prior studies and theoretical frameworks linking the vigilance decrement to mind wandering, declines in behavioral performance over time were predicted to covary with increases in mind wandering. In addition, greater motivation, interest, and perceived difficulty were hypothesized to buffer against these effects.

## 2 Methods

### 2.1 Participants

The study was approved by the University of Miami Institutional Review Board. Participants (*N* = 350; 58.3% female; mean age = 19.07 years, *SD* = 1.31) were recruited from the undergraduate psychology research pool via Sona Systems, provided informed consent, and received course research credit for their participation. A final sample of 310 participants (58.7% female; mean age = 19.06 years, *SD* = 1.31) were included in analyses post-exclusion (as detailed below). In our final sample, 309 participants provided demographic information regarding ethnicity and race. 66.99% of participants reported not a Hispanic or Latino ethnicity, 25.24% of participants reported a Hispanic or Latino ethnicity, and 7.77% preferred not to report ethnicity. Additionally, 64.40% of participants reported a White racial identity, 16.18% reported a Black racial identity, 13.27% reported an Asian racial identity, 0.65% reported a Native Hawaiian or Pacific Islander racial identity, 0.32% reported an American Indian or Alaskan Native racial identity, and the remaining 5.18% preferred not to report their racial identity.

### 2.2 Procedure

The study was conducted online using a web-based platform for data collection known as Inquisit Web (Millisecond Software, LLC). To help offset the impact of environmental distractions on task performance, participants were instructed at the onset of the study to find a quiet setting with a stable internet connection to complete a 30-min testing battery on a computer. The battery consisted of a series of self-report questionnaires followed by a modified 10-min version of the Sustained Attention to Response Task (SART; Robertson et al., 1997). The questionnaires assessed constructs related to wellbeing, personal memories and intrusions, and emotion regulation strategies. As these measures are beyond the scope of this brief report, they are not described or discussed further.

### 2.3 Sustained Attention to Response Task

The SART used in this study is modified from versions previously detailed by Zanesco et al. (2024a). Participants were shown a series of single digits (0–9) on screen, each appearing for 250 ms in black text on a white background. Participants were instructed to press the spacebar for all other digits (i.e., non-target trials) but withhold their response when the digit “3” occurred (i.e., target trials). Target trials occurred infrequently (~5%) relative to non-targets (15 target trials and 295 non-target trials). Throughout the SART, experience sampling mind wandering probes were presented intermittently (15 probes in total). Target and probe trials were distributed in a quasi-random order, such that there was a minimum of 5 non-targets before each target and 8 non-targets before each probe. The sequence of trials was identical for all participants. Participants began the SART by completing a practice block (79 trials), which was not included in analyses. The task took roughly 10 min to complete.

Each mind wandering probe asked two questions: The first probe asked, “where was your attention focused just before this question?” and responses ranged from 1 (“completely on-task”) to 5 (“completely off-task”); the second probe asked participants to “please characterize what you were thinking about just before this question?” and responses were selected from 1 of 6 categories, including (1 = “I was totally focused on the current task”, 2 = “I thought about my performance on the task”, 3 = “I was distracted by sights/sounds/physical sensations”, 4 = “I had negative thoughts unrelated to the task”, 5 = “I had positive thoughts unrelated to the task”, 6 = “I had neutral thoughts unrelated to the task”). On average, participants took 7.43 s to respond to both questions (*SD* = 17.24 s). Finally, upon completing the SART, participants were asked to report their felt motivation (“how motivated were you to do well on the task?”), interest (“how interested were you in the task?”), and difficulty (“how difficult was the task?”) they experienced while completing the task using a 1 (“not at all”) to 9 (“very”) point scale.

Two dependent measures describing task performance were calculated from behavioral responses in the SART. First, task accuracy was calculated using *A*′, a measure of signal detection sensitivity, (Stanislaw and Todorov, 1999), which accounts for accuracy on both non-target and target trials and is easy to calculate even with extreme hit and false alarm rates. While *A*′ is formally a measure of sensitivity in signal detection theory, it is conceptualized herein as a refined index of task accuracy—capturing the ability to correctly discriminate signal from noise while accounting for false alarms. Given potential issues with using *A*′ (Verde et al., 2006), we repeated the analyses using *d*′ and target accuracy. These analyses are reported in full in the Supplemental materials. In brief, the analyses for both metrics are consistent with the *A*′ results. Second, response time intra-individual coefficient of variation (RT ICV) was calculated as a measure of variability in response times (RT) by dividing individuals' standard deviation of RTs by their mean RT. Lastly, a dependent measure describing individuals' depth of mind wandering was obtained from ratings of attentional focus in the first probe question. We did not utilize ratings from the second probe question.

### 2.4 Analyses

In line with Zanesco et al. (2024a), we used bivariate growth curve modeling to examine within-task changes in attentional performance (*A*′ and RT ICV) and mind wandering over time-on-task and the covariance between changes in these metrics. We conducted two sets of analyses: a bivariate growth curve model examining *A*′ and mind wandering, and another examining RT ICV and mind wandering. Bivariate growth curve models describe the starting point (i.e., intercept) and linear rate of change (i.e., slope) for the two dependent measures within the same model as well as their slope covariance.

For the first bivariate growth curve model, mean *A*′ and mind wandering ratings were calculated for 3 consecutive blocks of trials. While the task ran continuously without breaks, blocks were constructed *post-hoc* so that target trials and mind wandering probes were distributed equally across blocks. Each block contained 5 target trials and 5 probes, as well as a comparable amount of non-target trials per block. Blocks with indeterminate *A*′ values were rare (1.4% of blocks across all participants) and excluded from analyses. *A*′ values were indeterminate when the participant either responds on every trial or when they did not respond on any trial, resulting in the hit rate and false alarm rate (1—non-target accuracy) to both be equal to 0 or both equal to 1. Fixed and random effects of block (intercept = first block) were included in the bivariate growth curve model of *A*′ and mind wandering to describe linear rates of change, variability, and between-person covariance in these metrics.

For the second bivariate growth curve model, RT ICV was calculated for sets of trials based on the seven non-target trials preceding each probe. These sets of non-target trials were uninterrupted by targets and the use of seven trials was consistent with numbers used in prior research (i.e., Zanesco et al., 2024a). RT ICV values were only included in analyses if there was 100% accuracy on non-targets preceding each probe. On average, participants had 14.65 (*SD* = 1.53, range = 2–15) probes that met this criterion. The corresponding mind wandering rating from each set of non-target trials was included as the second dependent variable in the growth curve model. Fixed and random effects of probe trials (intercept = first probe and corresponding preceding non-target trials) were added to this bivariate growth curve model to describe linear rates of change, variability, and between-person covariance in these metrics.

Finally, self-reported motivation, interest, and difficulty ratings were added to both bivariate growth curve models and examined as person-level moderators of within-task change in *A*′, RT ICV, and mind wandering. Seven participants were excluded from all analyses because they did not complete or prematurely ended the SART, 12 participants were excluded for not following task instructions (i.e., responding to < 66% of non-target trials), and 21 were excluded for poor overall performance (i.e., overall *A*′ was below 0.5). A final sample of 310 participants were included in analyses post-exclusion.

## 3 Results

Descriptives statistics describing overall performance and mind wandering in the Sustained Attention to Response Task (SART) align with prior studies (i.e., Zanesco et al., 2024a): mean *A*′ = 0.84 (*SD* = 0.09), mean RT ICV = 0.40 (*SD* = 0.20), and mean mind wandering ratings = 1.63 (*SD* = 0.57). Correlations between these task-averaged measures also confirm previously reported associations. *A*′ showed a negative correlation with RT ICV (*r* = −0.731, *p* < 0.001) and mind wandering ratings (*r* = −0.229, *p* < 0.001). Additionally, RT ICV was positively correlated with mind wandering ratings (*r* = 0.221, *p* < 0.001). On average, individuals with higher mind wandering ratings had lower *A*′ and greater RT ICV. Results for the bivariate models are described below and in Tables 1, 2.

**Table 1:** Accuracy *(A')* and mind wandering.

**Model effects**	**Estimate (*SE*)**
**Fixed effects**	
*A′* intercept_1_	0.8245 (0.0099)^***^
*A′* slope_1_	−0.0285 (0.0066)^***^
Mind wandering intercept_2_	1.442 (0.0336)^***^
Mind wandering slope_2_	0.1887 (0.0248)^***^
**Random effects**	
Intercept_1_ variance	−0.0031 (0.0037)
Intercept_2_ variance	0.2191 (0.03)^***^
Slope_1_ variance	−0.0065 (0.0020)^***^
Slope_2_ variance	0.1122 (0.0166)^***^
Intercept_1_ intercept_2_ covariance	−0.0151 (0.0059)^*^
Slope_1_ slope_2_ covariance	−0.0129 (0.0030)^***^
Intercept_1_ slope_1_ covariance	0.0086 (0.0021)^***^
Intercept_2_ slope_2_ covariance	−0.0313 (0.0172)
Intercept_1_ slope_2_ covariance	0.0039 (0.0044)
Intercept_2_ slope_1_ covariance	0.0092 (0.0040)^*^
Residual variance_1_	0.0397 (0.0033)^***^
Residual variance_2_	0.1571 (0.0126)^***^
Obs.	1,834
*N*	310

Parameter estimates from the bivariate model of SART accuracy (*A*′) and mind wandering. Fixed and random intercept and slope parameters are provided for each dependent measure in the model. Subscripts denote parameters for different dependent measures. Participants (*N*) and the number of observations (Obs.) included in the analysis are provided.

^*^p < 0.05.

^**^p < 0.01.

^***^p < 0.001.

**Table 2:** Response time variability (RT ICV) and mind wandering.

**Model effects**	**Estimate (*SE*)**
**Fixed effects**	
RT ICV intercept_1_	0.1929 (0.0074)^***^
RT ICV slope_1_	0.0061 (0.0007)^***^
Mind wandering intercept_2_	1.3424 (0.0367)^***^
Mind wandering slope_2_	0.0350 (0.0047)^***^
**Random effects**	
Intercept_1_ variance	0.0109 (0.0014)^***^
Intercept_2_ variance	0.2893 (0.034)^***^
Slope_1_ variance	0.00007 (0.00001)^***^
Slope_2_ variance	0.0049 (0.0006)^***^
Intercept_1_ intercept_2_ covariance	0.0081 (0.0049)
Slope_1_ slope_2_ covariance	0.0002 (0.00006)^***^
Intercept_1_ slope_1_ covariance	−0.0001 (0.0001)
Intercept_2_ slope_2_ covariance	−0.0196 (0.0037)^***^
Intercept_1_ slope_2_ covariance	−0.0007 (0.0006)
Intercept_2_ slope_1_ covariance	−0.0013 (0.0005)^**^
Residual variance_1_	0.0223 (0.0005)^***^
Residual variance_2_	0.4896 (0.0117)^***^
Obs.	8,338
*N*	310

Parameter estimates from the bivariate model of SART response time variability (RT ICV) and mind wandering. Fixed and random intercept and slope parameters are provided for each dependent measure in the model. Subscripts denote parameters for different dependent measures. Participants (*N*) and the number of observations (Obs.) included in the analysis are provided.

^*^p < 0.05.

^**^p < 0.01.

^***^p < 0.001.

### 3.1 Accuracy (*A′*)

**Figure 1:**
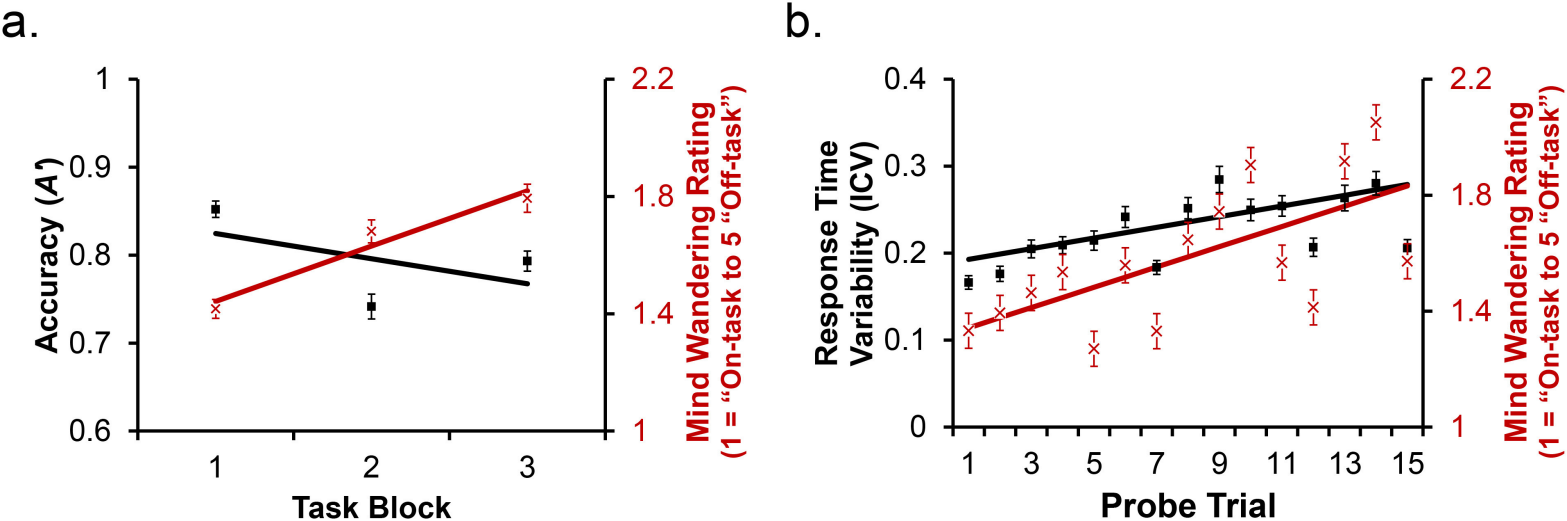
Bivariate growth curve models of performance and mind wandering over time-on-task. **(a)** Accuracy (*A*′) shown in black and mind wandering shown in red are plotted as a function of time-on-task across 3 task blocks. **(b)** Response time variability (RT ICV) shown in black and mind wandering shown in red are plotted as a function of time-on-task across 15 probe trials. Predicted model values are depicted as linear trend lines. Observed mean and standard error bars were calculated from subject averages at each block and probe trial.

The bivariate growth curve model revealed that *A*′ decreased linearly per block by −0.0285 units (*SE* = 0.0066, *p* < 0.001, 95% CI [−0.0416, −0.0155]) from the start of the task (*b* = 0.8245, *SE* = 0.0099, *p* < 0.001, 95% CI [0.8050, 0.8441]), indicating a 6.92% decrease in *A*′ from the first to the last block. Mind wandering increased linearly per block by 0.1887 units (*SE* = 0.0248, *p* < 0.001, 95% CI [0.1399, 0.2375]) from the start of the task (*b* = 1.4420, *SE* = 0.0336, *p* < 0.001, 95% CI [1.3759, 1.5082]), corresponding to a 26.17% increase in mind wandering from the first to the last block. Importantly, within-task changes in *A*′ and mind wandering were negatively associated (*r* = −0.4769, σ_slope1, slope2_ = −0.0129, *SE* = 0.0030, *p* < 0.001), as evidenced by the random effects covariance between slope parameters. These findings demonstrate that performance decrements in accuracy, as seen in the vigilance decrement, are associated with increased mind wandering over time-on-task (see Figure 1a).

### 3.2 Response time variability (RT ICV)

The bivariate growth curve model revealed that RT ICV increased linearly per probe by 0.0061 units (*SE* = 0.0007, *p* < 0.001, 95% CI [0.0047, 0.0076]) from the start of the task (*b* = 0.1929, *SE* = 0.0074, *p* < 0.001, 95% CI [0.1784, 0.2073]), representing a 44.56% increase in RT ICV from the first to the last probe. Mind wandering also increased linearly per probe by 0.0350 units (*SE* = 0.0047, *p* < 0.001, 95% CI [0.0257, 0.0443]) from the start of the task (*b* = 1.3424, *SE* = 0.0367, *p* < 0.001, 95% CI [1.2701, 1.4147]), corresponding to a 36.37% increase in mind wandering from the first to the last probe. Critically, within-task changes in RT ICV and mind wandering covaried positively (*r* = 0.380, σ_slope1, slope2_ = 0.0002, *SE* = 0.00006, *p* < 0.001), as evidenced by the random effects covariance between slope parameters. These findings show that within-task increases in RT ICV are associated with increased mind wandering over time-on-task (see Figure 1b).

### 3.3 Self-reported motivation, interest, and difficulty

We next added self-reported ratings of motivation, interest, and difficulty to both bivariate models as person-level moderators of within-task change. Overall, participants reported moderate levels of motivation (*M* = 6.47, *SD* = 2.59), interest (*M* = 5.14, *SD* = 2.75), and difficulty (*M* = 4.92, *SD* = 2.48) completing the task. We focus on reporting the effects on within-task changes below, while all results, including the effects at the start of the task, are detailed in Table 3.

**Table 3:** Motivation, interest, and difficulty bivariate growth curve models.

**Model effects**	**Intercept**	**Slope**	**Intercept**	**Slope**
	**Accuracy (** ***A'*** **)**		**Mind wandering**	
Motivation	0.0074 (0.0038)^*^	0.0037 (0.0025)	−0.0472 (0.0127)^***^	−0.0421 (0.0093)^***^
Interest	0.0047 (0.0036)	0.0038 (0.0024)	−0.0454 (0.0120)^***^	−0.0378 (0.0089)^***^
Difficulty	−0.0155 (0.0039)^***^	0.0036 (0.0027)	0.0059 (0.0135)	−0.0212 (0.0100)^*^
	**Response time variability (RT ICV)**		**Mind wandering**	
Motivation	−0.0064 (0.0028)^*^	−0.0008 (0.0003)^**^	−0.0259 (0.0141)	−0.0066 (0.0018)^***^
Interest	−0.0067 (0.0027)^*^	−0.0006 (0.0003)^*^	−0.0295 (0.0133)^*^	−0.0059 (0.0017)^***^
Difficulty	0.0039 (0.0030)	−0.0005 (0.0003)	0.0177 (0.0148)	−0.0048 (0.0019)^*^

Parameter estimates from predictors of intercept and slope estimates of SART accuracy (*A*′) and mind wandering (top) and response time variability (RT ICV) and mind wandering (bottom).

^*^p < 0.05.

^**^p < 0.01.

^***^p < 0.001.

Results from the bivariate model of *A*′ and mind wandering revealed that individuals who reported greater motivation, interest, and difficulty had less of a per-block increase in mind wandering, but no effect on *A*′. Similarly, results from the bivariate model of RT ICV and mind wandering revealed that individuals who reported greater motivation, interest, and difficulty had less of a per-probe increase in mind wandering. Additionally, those who reported greater task motivation and interest also had less of a per-probe increase in RT ICV. Together, these results show that task motivation and interest were consistently associated with lower RT ICV and mind wandering throughout the task.

## 4 Discussion

In the present study, we investigated whether declines in behavioral task performance are related to an increase in mind wandering during a short-form variant of a continuous performance task delivered online. Using bivariate growth curve models, we estimated linear within-task changes in accuracy (*A*′), response time variability (RT ICV), and probe-caught mind wandering as a function of time-on-task, as well as the covariance between these time-on-task effects. While prior studies have demonstrated robust associations between decrements in performance and increasing rates of mind wandering in longer tasks (e.g., 20 min: Zanesco et al., 2024a), these links have not been confirmed in shorter variants (e.g., 10 min). Specifically, we found that *A*′ declined over time-on-task, whereas RT ICV and rates of mind wandering increased as the task progressed. In line with prior studies (Zanesco et al., 2024a), within-task increases in mind wandering were directly associated with patterns of worsening performance and increasing response time variability. In addition, individual differences in self-reported task motivation and interest were consistently associated with within-task changes in response time variability and mind wandering, in line with prior research (Zanesco et al., 2024a). The current study also extended prior work by investigating these phenomena in a young adult sample of undergraduates who completed the task as part of an online assessment battery. These findings and their implications are discussed below.

Covariance between within-task changes in performance and mind wandering supports the view that mind wandering is a key factor contributing to performance declines with greater time-on-task. Establishing that mind wandering plays a significant role in the vigilance decrement suggests that the allocation of cognitive control to support task engagement may shift over time. One perspective, the resource control model (Thomson et al., 2015), posits that cognitive control diminishes over time, weakening the ability to maintain task goals. As cognitive control declines, attention increasingly drifts toward default cognitive patterns such as self-related thinking, a prominent content domain of mind wandering (Thomson et al., 2015; but see Smith et al., 2023). Another perspective, the opportunity cost model (Kurzban et al., 2013), suggests that as time spent on an unrewarding task increases, the perceived value of maintaining engagement diminishes relative to the benefits of allocating cognitive control to more rewarding alternatives. From this view, mind wandering increases over time as cognitive control shifts away from the task-at-hand and toward engagement with putatively more rewarding internally generated, off-task thoughts. Although these models offer distinct explanations—one attributing increased mind wandering to a loss of cognitive control and the other to its strategic reallocation—both predict a decline in performance and a rise in mind wandering with greater time-on-task.

Since both models predict performance declines and increased mind wandering with greater time-on-task, the observed covariance between performance and mind wandering does not distinguish between them. To clarify their relative contributions, future research could employ experimental manipulations to more precisely examine the underlying mechanisms. For example, systematically increasing cognitive load could test whether higher load not only accelerates performance declines, as predicted by the resource control model, but also increases mind wandering over time. Alternatively, the perceived reward value of the task could be adjusted to assess whether reduced reward accelerates mind wandering over time. In line with this prediction, prior research has demonstrated that reward manipulations and performance feedback can mitigate the vigilance decrement (Drody et al., 2025; Esterman et al., 2016; Hopstaken et al., 2015; Robison and Nguyen, 2023).

Although the present study did not experimentally manipulate such factors, an individual differences approach revealed that higher task motivation and interest were associated with both reduced performance costs, as indexed by response time variability, and lower levels of mind wandering over time. These findings suggest that individuals who assign greater subjective value to the task are more resilient to both the vigilance decrement and increases in mind wandering with time-on-task. One possible explanation, offered by the *expected value of control framework*, is that enhanced motivation dynamically adjusts the perceived costs and benefits of exerting control, thereby sustaining performance over time (Shenhav et al., 2013). Therefore, the current results align with growing evidence (see Brosowsky et al., 2023; Robison and Nguyen, 2023), support theories that motivation modulates the top-down control of attention (Shenhav et al., 2013), and highlight the practical relevance of motivation and sustained attention in academic and occupational settings.

While prior research has indicated that the vigilance decrement is reliably induced within the first 10 min of continuous performance tasks (See et al., 1995), it is possible that performance decrements in shorter variants of tasks represent different psychological processes than decrements in longer tasks, such as motivational, cognitive, or arousal processes (Thomson et al., 2015; Kurzban et al., 2013). However, our findings replicate patterns reported for longer variants of the Sustained Attention to Response Task (SART; i.e., Zanesco et al., 2024a) and confirm the validity of shorter variants for use in studies investigating mind wandering and the vigilance decrement. Specifically, the results indicate that over just 10 min, sustained attention begins to decline, and this decline is associated with an increase in mind wandering.

Furthermore, in addition to confirming the relationship between within-task performance decline and mind wandering, this study provides evidence that this link is evident not only in shorter tasks but also in tasks administered online. These findings are particularly relevant to academic settings, especially in the post-COVID era, where online learning modules have become more prevalent. Therefore, short online variants of cognitive tasks can be advantageous because they are more easily included in abbreviated assessment batteries in studies with time demands constraining the duration of individuals' research participation.

Overall, this study highlights the value of growth curve modeling approaches for examining the vigilance decrement and the inter- and intra- individual psychological factors that contribute to failures in sustained attention. One limitation of investigating vigilance and mind wandering in a short variant of SART, however, is that fewer trials necessarily contribute to aggregated measures of performance accuracy, such as the signal detection measure *A*′, calculated for blocks of the task. Estimates of change in *A*′ across blocks of the task should therefore be less reliable than if more trials contributed to aggregated measures in longer tasks. Future studies will need to directly evaluate the reliability of estimates of vigilance from shorter tasks relative to longer tasks in the same samples of individuals. Relatedly, the shorter duration of the task may have contributed to the low levels of self-reported mind wandering. Nevertheless, the initial levels of mind wandering and the pattern of change over time were consistent with findings from studies using longer tasks (Zanesco et al., 2024a,b). However, shorter duration tasks may limit the ability to detect subtle fluctuations over time between types of attentional lapses (e.g., spontaneous vs. deliberate mind wandering), which may be more likely to emerge during longer SART versions (Martinez-Perez et al., 2021). Collectively, these findings suggest that task duration should be guided by the research question—studies aiming to capture general patterns of mind wandering across participants may benefit from shorter tasks, while those seeking to differentiate between types of attentional lapses over time may require longer task versions.

Further, given that the SART was administered after a series of self-report questionnaires, there is the possibility of carry-over effects on SART outcomes. However, since this brief report focuses on the relationship between performance and mind wandering—not their absolute levels—such effects are less central to our interpretation. Future research should consider examining these influences to further assess the robustness and generalizability of these findings. Additionally, although participants in this study took only about 7 s on average to respond to both probe questions, the inclusion of intermittent mind wandering probes may have introduced brief pauses in the task progression which could have been served as rest-like periods or possibly induced task-switching effects. Such factors, along with the number of probes, could potentially influence mind wandering rates or task performance (He and Li, 2023; Greve and Was, 2022). Therefore, future studies employing more frequent probes or requiring longer response times may be better suited to investigate if and how such interruptions may affect performance or engagement.

Finally, not only do the current findings implicate mind wandering in the vigilance decrement, they also point to a practical avenue for mitigating performance declines in tasks requiring sustained attention. By establishing the link between the vigilance decrement and mind wandering, interventions known to reduce mind wandering, such as mindfulness training (see Jha et al., 2016), may provide a viable strategy for promoting sustained attention. This is particularly relevant for populations that must sustain performance in monotonous tasks where external task features cannot be easily adjusted to enhance engagement or where intrinsic motivation is low. In addition to its relevance for students in educational settings, targeted interventions to reduce mind wandering may benefit professionals in high-stakes environments, such as military personnel, air traffic controllers, and medical staff, by helping to prevent performance failures. These interventions may also benefit clinical populations, such as those with Attention-Deficit Hyperactivity Disorder, who report greater mind wandering both in daily life (Takim and Gokcay, 2025) and during attention tasks (Bozhilova et al., 2021). Future research should incorporate neural markers of cognitive control and attentional lapses to better elucidate the mechanisms underlying the correspondence between mind wandering and the vigilance decrement.

## Data Availability

The data and the analysis scripts supporting the conclusions of this article are available on OSF here: https://osf.io/rwjg2/.
