# Supplementary material for: Examining the association between vigilance and mind wandering
Source: Front Cognit. 2025 Sep 1;4:1577053. doi: 10.3389/fcogn.2025.1577053 (PMC13271118; doi:10.3389/fcogn.2025.1577053)
Supplement: Supplementary file 1 [file Data_Sheet_1.pdf]

Supplemental.

Given potential issues with A' (Verde et al., 2006), we conducted two additional sets of analyses to ensure that the observed trends in accuracy are replicated in other metrics. Overall, results from both models replicated the original results with A' in the manuscript.

We ran a bivariate growth curve model examining target accuracy and mind wandering, and another examining d' and mind wandering. Target accuracy measured participants' ability to correctly withhold responses to target trials. d' is a sensitivity metric from signal detection theory (Stanislaw and Todorov, 1999). To correct for indeterminate d' values, we used log-linear correction of hit and false alarm rates (Hautus, 1995).

$$Hit\ Rate\ transformed = \frac{\#\ of\ Hits + .5}{\#\ of\ Targets + 1}$$

$$False\ Alarm\ Rate\ transformed = \frac{\#\ of\ False\ Alarms + .5}{\#\ of\ Non\text{-}Targets + 1}$$

$$d' = z(\mathrm{H}) - z(FA)$$

Mean target accuracy and mind wandering ratings were calculated for three consecutive blocks of trials were entered for the first model, and mean d' and mind wandering ratings were calculated for three consecutive blocks of trials were entered for the second model. Results for the bivariate models are described below and in Tables 1 and 2.

Target Accuracy.

The bivariate growth curve model revealed that target accuracy decreased linearly by –0.0416 units (SE = 0.0091, p < .001, 95% CI [–0.0595, –0.0237]) each block from the start of the task (b = 0.4982, SE = 0.0160, p < .001, 95% CI [0.4666, 0.5297]), indicating a 16.70% decrease in target accuracy from the first to the last block. Mind wandering increased linearly per block by 0.1887 units (SE = 0.0248, p < .001, 95% CI [0.1399, 0.2375]) from the start of the task (b =

1.4420, *SE* = 0.0336, $p < .001$, 95% CI [1.3759, 1.5082]), corresponding to a 26.17% increase in mind wandering from the first to the last block. Importantly, within-task changes in target accuracy and mind wandering were negatively associated (r = –0.2468, $\sigma_{slope1, slope2}$ = –0.0163, SE = 0.0041, $p < .001$), as evidenced by the random effects covariance between slope parameters. These findings demonstrate that performance decrements in accuracy, as seen in the vigilance decrement, are associated with increased mind wandering over time-on-task (see Figure 1a).

d′

The bivariate growth curve model revealed that *d′* decreased linearly by –0.2165 units (*SE* = 0.0280, $p < .001$, 95% CI [–0.2717, –0.1614]) each block from the start of the task (*b* = 2.5185, *SE* = 0.0467, $p < .001$, 95% CI [2.4267, 2.6104]), indicating a 17.19% decrease in *d′* from the first to the last block. Mind wandering increased linearly per block by 0.1887 units (*SE* = 0.0248, $p < .001$, 95% CI [0.1399, 0.2375]) from the start of the task (*b* = 1.4420, *SE* = 0.0336, $p < .001$, 95% CI [1.3759, 1.5082]), corresponding to a 26.17% increase in mind wandering from the first to the last block. Importantly, within-task changes in *d′* and mind wandering were negatively associated (r = –0.7347, $\sigma_{slope1, slope2}$ = –0.0657, SE = 0.0128, $p < .001$), as evidenced by the random effects covariance between slope parameters. These findings demonstrate that performance decrements in accuracy, as seen in the vigilance decrement, are associated with increased mind wandering over time-on-task (see Figure 2a).

Self-reported motivation, interest, and difficulty

We also repeated our analyses adding self-reported ratings of motivation, interest, and difficulty to both bivariate models above as person-level moderators of within-task change (see Table 3). Consistent with original findings, individuals who reported greater motivation, interest,

and difficulty had less of a per-block increase in mind wandering across both models. While there were no effects of motivation, interest, and difficulty on *A'* in the manuscript, slope effects emerged here. Specifically, those who reported greater motivation, interest, and difficulty had less of a per-block decrease in *d'*. Lastly, those who reported greater difficulty had less of a per-block decrease in target accuracy.

References

Hautus, M. J. (1995). Corrections for extreme proportions and their biasing effects on estimated values of d'. *Behavior Research Methods, Instruments, & Computers*, 27, 46-51.

Stanislaw, H., & Todorov, N. (1999). Calculation of signal detection theory measures. *Behavior Research Methods, Instruments, & Computers*, 137-149.

Verde, M. F., Macmillan, N. A., & Rotello, C. M. (2006). Measures of sensitivity based on a single hit rate and false alarm rate: The accuracy, precision, and robustness of d', Az, and A'. *Perception & Psychophysics*, 68, 643-654.

Table 1.

Target Accuracy and Mind Wandering

Model Effects	Estimate (*SE*)
Fixed Effects	
Target Accuracy Intercept1	0.4982 (0.016)***
Target Accuracy Slope1	-0.0416 (0.0091)***
Mind Wandering Intercept_2	1.442 (0.0336)***
Mind Wandering Slope_2	0.1887 (0.0248)***
Random Effects	
Intercept_1 variance	0.0357 (0.0073)***
Intercept_2 variance	0.2191 (0.03)***
Slope_1 variance	-0.0007 (0.003)
Slope_2 variance	0.1122 (0.0166)***
Intercept_1 Intercept_2 covariance	-0.0206 (0.0096)*
Slope_1 Slope_2 covariance	-0.0163 (0.0041)***
Intercept_1 Slope_1 covariance	-0.0006 (0.0037)
Intercept_2 Slope_2 covariance	-0.0313 (0.0172)
Intercept_1 Slope_2 covariance	0.0068 (0.007)
Intercept_2 Slope_1 covariance	0.0137 (0.0054)*
Residual variance_1	0.1571 (0.0126)***
Residual variance_2	0.0527 (0.0042)***
Obs.	1860
N	*310*

Note. Parameter estimates from the bivariate model of SART target accuracy and mind wandering. Fixed and random intercept and slope parameters are provided for each dependent measure in the model. Subscripts denote parameters for different dependent measures. Participants (N) and the number of observations (Obs.) included in the analysis are provided. * = $p < .05$, ** = $p < .01$, *** = $p < .001$.

Table 2.
d′ and Mind Wandering

Model Effects	Estimate (*SE*)
Fixed Effects	
d′ Intercept_1	2.5185 (0.0467)***
d′ Slope_1	-0.2165 (0.0280)***
Mind Wandering Intercept_2	1.4420 (0.0336)***
Mind Wandering Slope_2	0.1887 (0.0248)***
Random Effects	
Intercept_1 variance	0.3887 (0.0590)***
Intercept_2 variance	0.2191 (0.0300)***
Slope_1 variance	0.0712 (0.0239)**
Slope_2 variance	0.1122 (0.0166)***
Intercept_1 Intercept_2 covariance	-0.0747 (0.0279)**
Slope_1 Slope_2 covariance	-0.0657 (0.0128)***
Intercept_1 Slope_1 covariance	-0.0249 (0.0291)
Intercept_2 Slope_2 covariance	-0.0313 (0.0172)
Intercept_1 Slope_2 covariance	0.0119 (0.0204)
Intercept_2 Slope_1 covariance	0.0291 (0.0167)
Residual variance_1	0.3442 (0.0276)***
Residual variance_2	0.1571 (0.0126)***
Obs.	1860
N	*310*

Note. Parameter estimates from the bivariate model of SART accuracy (*d′*) and mind wandering. Fixed and random intercept and slope parameters are provided for each dependent measure in the model. Subscripts denote parameters for different dependent measures. Participants (N) and the number of observations (Obs.) included in the analysis are provided. * = $p < .05$, ** = $p < .01$, *** = $p < .001$.

Table 3.
Motivation, Interest, and Difficulty Bivariate Growth Curve Models

	Intercept	Slope	Intercept	Slope
Model Effects	Target Accuracy		Mind Wandering	
Motivation	0.0054 (0.0062)	0.0038 (0.0035)	-0.0472 (0.0127)***	-0.0421 (0.0093)***
Interest	0.0009 (0.0058)	0.0052 (0.0033)	-0.0454 (0.0120)***	-0.0378 (0.0089)***
Difficulty	-0.0344 (0.0062)***	0.0078 (0.0036)*	0.0059 (0.0135)	-0.0212 (0.0100)*
	d′		Mind Wandering	
Motivation	0.0566 (0.0176)**	0.0222 (0.0107)*	-0.0472 (0.0127)***	-0.0421 (0.0093)***
Interest	0.0329 (0.0120)	0.0218 (0.0102)*	-0.0454 (0.0120)***	-0.0378 (0.0089)***
Difficulty	-0.0790 (0.0182)***	0.0255 (0.0112)*	0.0059 (0.0135)	-0.0212 (0.0100)*

Note. Parameter estimates from predictors of intercept and slope estimates of SART target accuracy and mind wandering (top) and *d′* and mind wandering (bottom). * = $p < .05$, ** = $p < .01$, *** = $p < .001$.

Figure 1.

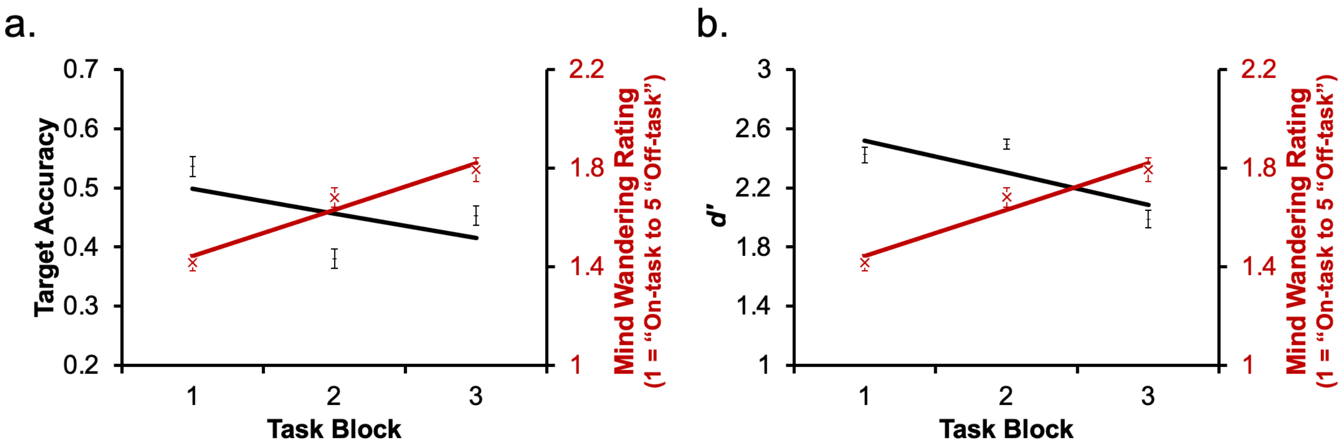

Note. Bivariate growth curve models of performance and mind wandering over time-on-task. (a) Target accuracy shown in black and mind wandering shown in red are plotted as a function of time-on-task across 3 task blocks. (b) *d′* shown in black and mind wandering shown in red are plotted as a function of time-on-task across 3 task blocks. Predicted model values are depicted as linear trend lines. Observed mean and standard error bars were calculated from subject averages at each block.
